# Phages and Nanotechnology: New Insights against Multidrug-Resistant Bacteria

**DOI:** 10.34133/bdr.0004

**Published:** 2023-01-16

**Authors:** Marco Pardo-Freire, Pilar Domingo-Calap

**Affiliations:** Institute for Integrative Systems Biology, I2SysBio, Universitat de València-CSIC, 46980 Paterna, Spain.

## Abstract

Bacterial infections are a major threat to the human healthcare system worldwide, as antibiotics are becoming less effective due to the emergence of multidrug-resistant strains. Therefore, there is a need to explore nontraditional antimicrobial alternatives to support rapid interventions and combat the spread of pathogenic bacteria. New nonantibiotic approaches are being developed, many of them at the interface of physics, nanotechnology, and microbiology. While physical factors (e.g., pressure, temperature, and ultraviolet light) are typically used in the sterilization process, nanoparticles and phages (bacterial viruses) are also applied to combat pathogenic bacteria. Particularly, phage-based therapies are rising due to the unparalleled specificity and high bactericidal activity of phages. Despite the success of phages mostly as compassionate use in clinical cases, some drawbacks need to be addressed, mainly related to their stability, bioavailability, and systemic administration. Combining phages with nanoparticles can improve their performance in vivo. Thus, the combination of nanotechnology and phages might provide tools for the rapid and accurate detection of bacteria in biological samples (diagnosis and typing), and the development of antimicrobials that combine the selectivity of phages with the efficacy of targeted therapy, such as photothermal ablation or photodynamic therapies. In this review, we aim to provide an overview of how phage-based nanotechnology represents a step forward in the fight against multidrug-resistant bacteria.

## Introduction

The emergence of multidrug-resistant (MDR) bacteria is the result of the misuse and overuse of antibiotics, which have led to the emergence of MDR strains worldwide, being a major health concern. In 2019, more than 1.2 million people died due to MDR bacteria [1] and it is expected that, by 2050, MDR bacteria will be the leading cause of death worldwide [2–4]. In the last 40 years, research and development for new antibiotics have decreased dramatically. Since 2017, only 11 new antibiotics have been approved, most of them being modifications of prior existing antibiotics, with only 2 of them representing a new chemical scaffold [5]. Under this scenario, novel strategies of control are mandatory to reduce the emergence and spread of MDR bacteria worldwide.

Bacteriophages or phages, viruses that infect bacteria, represent a promising tool against MDR bacteria. Phages are the most abundant entity in the biosphere, with more than 10^31^ viral particles around the globe [6]. Lytic phages are the most valuable to treat or prevent bacterial infections since they kill bacteria and are not able to transfer resistance genes horizontally [7]. This approach, known as phage therapy, consists in the use of phages as therapeutic tools against MDR bacteria. Phages are highly specific, usually having a narrow host range, with no effect on nontargeted bacteria, avoiding dysbiosis and reducing ecological impact. In addition, phages multiply at the site of infection, but in the absence of their bacterial host, they are degraded. Although phages can induce bacterial resistance, they coevolve with the bacteria and can overcome resistance. Moreover, it has been shown that the resistance to a phage may resensitize bacteria to antibiotics or become avirulent [7]. Yet, the inherent characteristics of phages also have some disadvantages concerning their clinical application. For example, due to their high specificity, the use of phages would require identification of the bacterial strain and may involve the need for phage hunting or phagograms before the treatment. This could be time consuming and difficult to achieve, especially in acute infections. For more effective treatments, phage cocktails including a combination of different phages to increase host range or delay resistance have been proposed [8]. Another solution is to evolve phages in vitro to train them to improve their host range or other physical–chemical properties [9]. In addition, phages are subject to protein misfolding, aggregation, or denaturation that leads to a loss of integrity and infectivity after exposure to certain harsh conditions, such as organic solvents, pH, temperature, and salinity [10–13]. Furthermore, in some cases, the low diffusion and penetration of phage particles into tissues make it difficult for them to reach the infection sites.

**Fig. 1.**
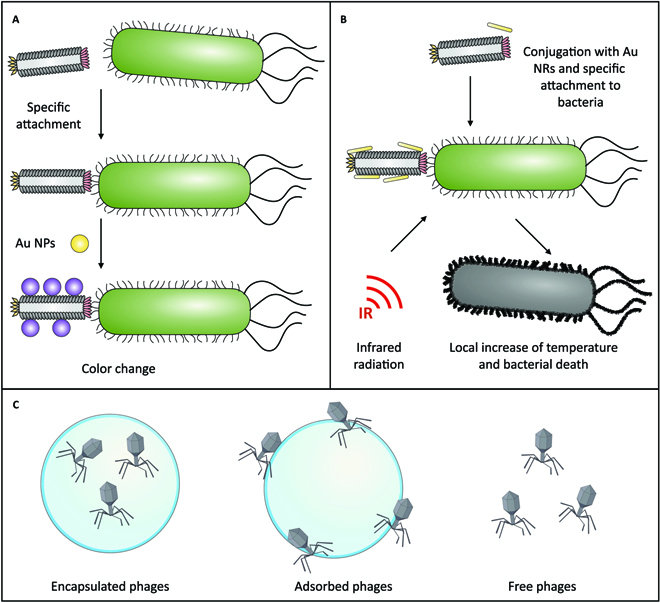
Examples of the combination of nanotechnology with phages. (A) Use of phages conjugated with gold NPs for the identification of bacteria (detection, sensing, typing) based on color changes due to their surface plasmon resonance properties. (B) Use of phages conjugated with gold nanorods (AuNRs) for focalized photothermal therapy. (C) Encapsulation and adsorption of phages in NPs and MPs for improved clinical output of phage therapy in contrast with free phage administration.

All these drawbacks give special interest to the development of alternative phage-based formulations [14]. The combination of phage therapy with nanotechnology, i.e., different types of nanoparticles (NPs) or microparticles (MPs), offers a reasonable way to improve the clinical output of phage therapy, from the design of rapid devices for bacterial identification to the development of platforms that enhances the bioavailability of phages, prevent their inactivation, provide shielding against the effects of neutralizing antibodies (Abs), reduce their rapid clearance by the reticuloendothelial system, enhance their stability during long-term storage, and provide targeted delivery and controlled phage release [15]. In this review, we aim to gather research on phage–NP-based biosensors for bacterial detection or typing, phage–NP-mediated focalized therapy, and the encapsulation of phages for improved phage therapy, with a special focus on in vivo studies (Fig. 1).

## Phage-Based Nanobiosensors for Bacterial Detection

The detection of pathogens from biological samples must be selective, sensitive, and fast to prevent and control infections and disease outbreaks. However, most methods available are time consuming or require advanced analytical equipment [16]. Culture methods are predominantly used for bacterial detection, but they require time for bacterial growth [16,17]. Mass spectroscopy and biochemical detection systems are faster but still have some drawbacks such as high cost and difficult handling. Polymerase chain reaction (PCR) and immunoassays are designed to improve the sensitivity of detection, but they are not able to distinguish between live and dead cells [18]. On the one hand, PCR detection requires DNA extractions that are time consuming and lack fidelity of DNA replication. On the other hand, immunoassays are simple and rapid, but their sensitivity may not be enough for pathogen detection [19,20]. Developing new sensors to detect and discriminate bacteria at low concentrations, quickly, and with high specificity and sensitivity is needed.

Nanotechnology can provide interesting biomedical applications based on nanomaterials such as the development of biosensors and probes for pathogen detection, using NPs as carriers of recognition elements (e.g., aptamers, Abs, enzymes, DNA, and phages) [21, 22]. In the case of bacterial detection, these recognition elements are responsible for the specific attachment of the target bacteria, forming an NP–bacteria complex that can be detected through color changes in the solution, NP aggregation, mass spectrometry peaks, electrochemical signals, and surface plasmon resonance (SPR) shift. Metallic NPs, in particular gold and silver NPs, are commonly used in bacterial detection taking advantage of their SPR properties [22, 23]. Plasmonic NPs include a recognition element (e.g., Ab, aptamer, or phage) that interacts with a bacterium, causing the aggregation of the NPs, a shift of the plasmonic peak, or a change in color [24–26]. These approaches based on color changes present advantages because they are rapid, simple, and not limited to laboratory settings.

The aggregation of NPs coated with Abs is widely used for the detection of bacteria [27]. Each Ab has a high affinity for a specific antigen expressed in bacteria, and the sensitivity can be improved depending on NP size, shape, binding site number, and aggregation state. Polyclonal Abs recognize epitopes that can be present in other nonpathogenic organisms related to the target bacterium, presenting low selectivity [28]. Monoclonal Abs can overcome this problem, recognizing bacteria with enough sensitivity and selectivity, but are relatively unstable to harsh environmental conditions, limiting their potential use and long-term storage. Furthermore, their production costs are too high.

Aptamers are tridimensional structures formed by the folding of a short sequence of amino acids, ribonucleotides, or deoxyribonucleotides. Depending on their sequence, they can detect a broad range of structures, including bacteria, with a high degree of affinity and specificity [29]. Nucleotide aptamer production is easier and cheaper compared to Abs. In addition, toxicity and immune responses have not been reported even at high doses. Aptamers are widely used as recognition elements, but they show poor stability in biological mediums and certain limitations due to the formation of secondary structures that can lead to reduced specificity by cross-reactivity [30].

Another possibility is the use of phages. However, only a few studies have described them as recognition elements, despite their advantages in bacterial detection. Phages can produce proteins that selectively attach to bacterial receptors, being able to distinguish between live and dead cells by monitoring viral replication or expression of reporter genes [31]. In contrast with Abs or aptamers, phages are easily produced at a very low cost and are more resistant to environmental stress (e.g., temperature or pH) [32]. Genetic engineering can be used to modify model phages to express receptor-binding proteins (RBPs) that target a desired bacterial species. As an example, the filamentous phage M13 was modified to express RBPs targeting several bacterial species (*Escherichia coli*, *Pseudomonas aeruginosa*, *Vibrio cholerae*, and *Xanthomonas campestris*) [33]. The engineered phages were thiolated to react with NPs, creating a phage–NP complex by means of a thiol bond. Once the modified phages are attached to their target bacteria, gold NPs are added to the medium to interact with phages, causing their aggregation and a change in the color of the solution, due to SPR peak shift, indicating the presence of the target bacteria in the medium. Another approximation was done by generating recombinant tail fiber (TF) proteins deriving from TF proteins of phages ɸAB2 and ɸAB6, aiming to detect *Acinetobacter baumannii* M3237 and 54149, respectively. In this case, the TF was combined with aluminum magnetic NPs through a hexahistidine tag. After the recognition of the bacteria by the complex, they were easily separated using a magnetic field and analyzed by mass spectrometry. The results showed that the TF–NPs were able to discriminate between the 2 *A. baumannii* strains tested, detecting them with high sensitivity [34]. In a different approach, SiO_2_@Au NPs were decorated with S13″ phage on their surface to form a plasmon-scattering nanoprobe. The phage can selectively bind *Staphylococcus aureus* in the presence of an excess of *E. coli*, while the plasmon-scattering NPs can be detected by dark-field microscopy. Since the binding of the bacteria by the phage causes the aggregation of the NPs, the scattering intensity around the target bacteria was higher than that caused by the random attachment of some probes to *E. coli* [35]. The abovementioned and other examples of phage-based biosensors are listed in Table 1. Phage-based assays may also be based on the expression of a reporter gene after phage infection of the target cells or the production of phages bearing a fluorescent or enzymatic tag [36–38]. Phages have also been used to identify *E. coli* and methicillin-resistant *S. aureus* conjugated to a gold surface, detecting changes in impedance [39], although these procedures require more time and specific equipment that may not be accessible in point-of-care settings for rapid detection. A promising approach could be the encapsulation of NPs inside the capsid of phages, avoiding NP interference in bacterial recognition, although this technology has not been used yet for bacterial detection [40]. However, the use of NP–phage-based biosensors for bacterial detection (diagnosis and typing) is still promising since it reduces the need for sample preparation and has high operational and storage stability, fast reaction time, easy manipulation, and portability.

**Table 1.** Phage-based nano/biosensors for bacterial detection and typing.

NP composition	Targetbacteria	Detection method	Recognition element	Advantages	NP size (nm)	Detection sensitivity	Reference (year)
Gold nanorods(AuNR)	*E. coli*	Color change/UV-Vis spectroscopy	T7 phage	Detection of β-galactose after bacterial lysis by T7 phage	Length: 59 ± 8Width: 11 ± 1	10^4^ CFU ml^−1^	103 (2016)
Gold NPs(AuNP)	*E. coli* *P. aeruginosa* *V. cholerae* *X. campestris*	Color change/UV-Vis spectroscopy	Chimeric M13 phage	Detection limit can be lowered by using a lower resuspension volumeNo cross-reactivity reported	8	100 cells	33 (2018)
Alumina-coated magnetic NPs	*A. baumannii*M3237 and 54149	Mass spectrometry	Tail fibers 2 and 6 displayed in ɸAB2 and ɸAB6 phages, respectively	Distinguished *A. baumannii* M3237 from *A. baumannii* 54149		*A. baumannii* M3237: 10^5^ CFU ml^−1^*A. baumannii* 54149: 10^4^ CFU ml^−1^	34 (2019)
SiO_2_@Au core–shell NPs	*S. aureus*	Dark-field microscopy imaging	Phage S13′	Specific detection of *S. aureus* in the presence of other bacteria such as*E. coli*	557	8 × 10^4^ CFU ml^−1^	35 (2019)
Albumin-templated Co_3_O_4_ magnetic nanozymes	*S. aureus*	Magnetophoretic chromatography and color change using the catalytic oxidation of ABTS	Phage fusion pVIIIprotein	Detection of *S. aureus* in complex media such as milk	210	8 CFU ml^−1^	104 (2020)

NP, nanoparticle; NR, nanorod (nanoscale object synthesized from metals or semiconducting materials in which the growth rate changes depending on the face, creating an elongated shape); core–shell, nanoparticle structure based on an inner core structure and an outer shell made of different components (e.g., 2 different metals, metal and polymer, or 2 different polymers or biopolymers); nanozymes, nanomaterials that replicate enzyme properties with higher stability and lower cost

## Phage- and NP-Mediated Focalized Therapy

NPs can be used to kill bacteria through localized hyperthermia [photothermal therapy (PTT)] and induction of oxidative stress [photodynamic therapy (PDT)] under near-infrared (NIR) irradiation [41,42]. The NIR radiation causes NP excitation with 2 possible outcomes: a release of energy through nonradiative decay pathways generating heat in PTT or interaction with oxygen by either electron or energy transfer to generate reactive oxygen species in PDT [43]. The 2 methods are noninvasive and can act with high selectivity and low toxicity. One of the major concerns in the clinical treatment of bacteria is the formation of biofilms. These structures are formed by cells and extracellular polymeric substances (EPSs) that confer protection to the bacteria and capacity of attachment to surfaces such as clinical surfaces, medical devices, and prostheses [44]. NPs have also been studied for the treatment of biofilms. In the case of PTT and PDT, NPs composed of Cu_9_S_8_ and covered with polyethylene glycol (PEG) attract particular interest since their properties suit both methodologies. It was demonstrated that this type of NPs was able to remove biofilms formed by *S. aureus* on titanium surfaces by a combination of both PTT and PDT [41]. One of the drawbacks of PTT is that cells or tissues surrounding the bacteria can reach local temperatures up to 50 °C [45], which can be damaging for them [42]. However, some works have reported successful in vivo assays in the employment of PDT with a low-temperature PTT (less than 45 °C) [46]. One requirement for a successful PTT or PDT treatment is that the NPs can effectively penetrate the EPS to reach the bacteria buried in the deep of the matrix; otherwise, the infection may become persistent as it has been shown for conventional antimicrobial treatments [47–48]. There are enzymes, such as deoxyribonuclease (DNase), that can disrupt some biofilms and thus prevent the colonization of bacteria on certain surfaces [49]. DNase has been used in conjugation with gold nanoclusters (DNase–AuNCs) for the treatment of biofilm-related infections. The enzymatic part of the complex allows the disruption of the EPS, allowing the AuNCs to reach the bacteria within the biofilm matrix where they are eradicated using PDT and PTT. This is an example of combined PDT and PTT therapy with enzymolysis that has proved to be effective against biofilms formed in orthodontic appliances by *S. aureus*, *Staphylococcus epidermidis*, *E. coli*, and *P. aeruginosa* [50].

In the case of phages, the association with PTT can be used to destroy the phages after recognition, reducing potential horizontal gene transfer or phage multiplication and evolution [51]. Although lytic phages are commonly used in phage therapy to avoid gene transduction, the use of PTT that destroys the phages after the recognition of the bacteria eliminates that possibility. One example of PTT-phage combined therapy was carried out by linking phages with gold nanorods (AuNR) to form a structure known as “phanorods.” This was achieved by the thiolation of M13 phage proteins in a manner that they could subsequently react with the surface of AuNR, forming a sulfur bond that kept the resulting phanorod together. Different chimeric M13 phages, targeting *E. coli*, *P. aeruginosa*, *V. cholerae*, and *X. campestris*, were designed and conjugated with AuNRs. The efficacy of the corresponding phanorods was demonstrated in an aqueous solution using PTT ablation for the 4 species. In the case of *P. aeruginosa*, the efficacy was also tested in biofilm growing on epithelial cells, causing bacterial death with little damage to the surrounding epithelium [51]. In clinics, phanorods are particularly attractive for the treatment of superficial infections such as wounds or in the disinfection of medical devices, where they can be applied to biofilms. Interestingly, these assays were extended to the treatment of wound infections in a mouse model [52]. Although the efficacy of phanorod–PTT combined therapy was observed in vitro, it was not clear if it will be maintained in vivo. The issues that may have arisen included immune system inhibition, unknown phanorod toxicity, or uncontrolled damage to surrounding cells or tissues. A chimeric M13 phage recognizing *P. aeruginosa* was conjugated with AuNRs as previously described, but in this case, a zinc-binding peptide was also attached to the complex. In this way, the phanorod also serves as a Zn^2+^ delivery system for bacterial infection. The activation of the AuNRs by NIR after phage–bacteria interaction would cause localized heating and cell death, as well as Zn^2+^ liberation on the infection site. The release of Zn^2+^ potentiates antibacterial activity and helps in wound healing [53,54]. This 3-part complex was evaluated in a mouse model, treating wounds infected with *P. aeruginosa*. The results showed that both phanorod and phanorod–Zn treatments were highly effective, yielding faster healing when compared with standard antibiotic treatments or antibiotics conjugated with AuNRs. Additionally, phanorod–Zn treatments were also effective when administered to late and severe infections and in wounds infected by MDR *P. aeruginosa*. No substantial toxicity biomarkers were found after phanorod or phanorod–Zn application. Different approaches have been evaluated, for example, the irradiation of AuNP-coated M13 phage irradiated by a 532-nm laser (visible light) that resulted in between 21% and 64% *E. coli* reduction in liquid culture [55]. These results indicate that phage-based nanomaterials, such as phanorods, may be a promising alternative antimicrobial strategy for the treatment of MDR bacterial localized infections or wounds.

## Encapsulation of Phages for Improved Phage Therapy

**Fig. 2.**
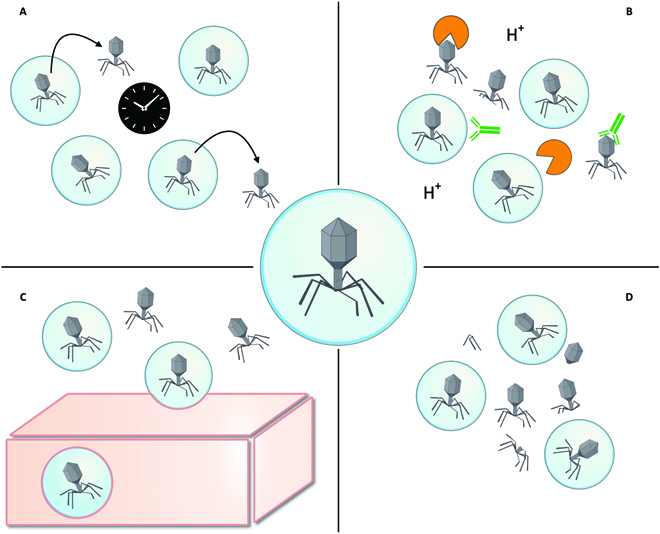
Properties of encapsulated phages that may improve phage therapy. (A) Controlled and prolonged release over time. (B) Protection against conditions that may inactivate phages such as acidic pH, neutralizing antibodies (green), and enzymes (orange). (C) Higher adhesion permeability and diffusion into tissues as compared to free phages by the interaction of the particle with the tissue. (D) Higher stability of phage particles during storage or administration as compared to free phages.

**Fig. 3.**
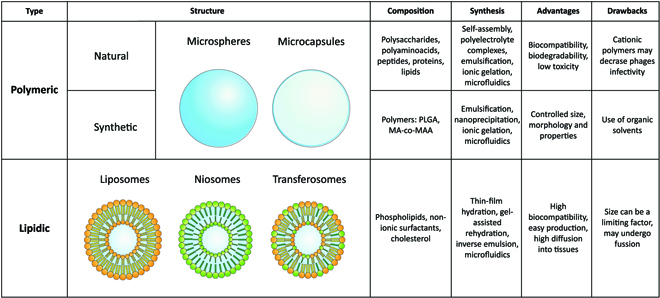
Main types of NPs (natural polymeric, synthetic polymeric, and lipidic) along with their possible structures: microspheres (solid), microcapsules (hollow), liposomes (phospholipids), niosomes (nonionic surfactants), and transferosomes (phospholipids and nonionic surfactants). Details on composition, methods of synthesis, and a list of their principal advantages and drawbacks are listed for each type of NP. PLGA, poly(lactic-co-glycolic) acid; MA-co-MAA, methacrylate-co-methacrylic acid-based.

Phage therapy has numerous advantages over traditional antibiotics, but this does not mean that they are the perfect treatment. Phages must deal with some obstacles when applied in vivo; these include low bioavailability, loss of activity caused by external factor such as temperature or pH changes, or elimination by the host immune system either by the reticuloendothelial system or by means of Abs. Encapsulation of therapeutic phages presents itself as the path for optimizing phage therapy, as both a delivery and protection system [56]. Some advantages of encapsulated phages are shown in Fig. 2. In general, most of the delivery systems suitable for phage therapy involve both natural and synthetic polymers and liposomes (Fig. 3). Each of them has different characteristics and follows different synthesis methods. Election of the appropriate system and production process depends on the required application and form of administration. The size, morphology, stability, and titer of encapsulated phages are other factors that vary among encapsulation methods and determine the efficacy of the final formulation [57,58]. It is impossible to design “one fit to all” platform for phage encapsulation, since the variability of phages and possible hosts is boundless and personalized approaches are needed. In the past, phage encapsulation has been mainly focused on the delivery of phages through the gastrointestinal tract. In this context, the use of polymeric NPs and MPs served to protect phages from low pH inactivation, digestive enzymes, or bile juices. More interestingly, they increased the permeability to mucus layer, reaching the site of residence of some bacterial pathogens. Encapsulation is also involved in the controlled release of the phage and allows protection against neutralizing Abs [57]. The number of studies in animal models is increasing in the last years and provides valuable knowledge of pharmacokinetic parameters for the development of new treatments for bacterial infections based on encapsulated phages. Several relevant studies both in vitro and in vivo are summarized in Table 2.

**Table 2.** Phage encapsulation methods—in vitro and in vivo studies.

Particlecomposition and size	Encapsulated phages	Bacteria	Administration/use	Animal model	Remark	Reference(Year)
Chitosan298 nm	Phage cocktail	*S. enterica* *S. flexneri* *E. coli*	Oral	Rat	Encapsulation protects phages at acidic pH	63 (2021)
Porous PLGA(w/o/w)14 μm	Recombinant fd expressing OVA antigen	*E. coli*	Vaccine development	None(in vitro)	Genetically engineered fd filamentous phage	86 (2020)
Porous PLGA(w/o/w)8 μm	Phage cocktail	*P. aeruginosa*	Inhalation of lyophilized powder formulation	Mice	Used in lung infections mimickingcystic fibrosis condition	81 (2018)
Eudragit L100100 μm	FGS011	*Salmonella* sp.	Oral	Broilers	Increase titer in cecum	79 (2020)
Eudragit S100/alginate100 μm	Felix O1	*E .coli*	N.A.	None(in vitro)	pH responsiveness	66 (2019)
Liposomes342 nm	UAB_Phi20	*S. enterica*	Oral	Mice	Phage transcytosis of intestinal barrierBiodistribution of phages in mice	90 (2019)
Liposomes100–300 nm	T3 (*E. coli*)K (*S. aureus*)	*E. coli* *S. aureus*	N.A.	None	Adjusted encapsulation yield	96 (2018)
Liposomes 135–218 nm (PEV2)261–448 nm (PEV40)	PEV2PEV40	*P. aeruginosa*	N.A.	None	Size variation between phages	95 (2018)
Liposomes100–300 nm	MR-5MR-10	*S. aureus*	Local	Mice	Diabetic wound infection	93 (2018)
Liposomes230 nm	Phage cocktail	*K. pneumoniae*	Local	Mice	Burn wound infection	92 (2017)
Liposomes309–326 nm	UAB_Phi20UAB_Phi78UAB_Phi87	*Salmonella* spp.	Oral	Broiler	Long-term efficacy	89 (2015)
Liposomes102 nm	KPO1K2	*K. pneumoniae*	N.A.	None(in vitro)	Shielding from neutralizing Abs.Intracellular delivery	100 (2016)
Liposomes150–700 nm	KPO1K2	*K. pneumoniae*	Systemic (injection)	Mice	Prolonged release and retainmentHigh biodistribution	91 (2016)

N.A., no available; Abs, antibodies; w/o/w, water-in-oil-in-water emulsion

### Natural polymers

Biopolymers are polymers formed by natural macromolecules. Due to their natural origin, they usually have good biocompatibility and biodegradability, and consequently, they are excellent for several biomedical applications, including phage encapsulation. NPs and MPs with characteristic sizes of 1 nm to 1 μm and 1 to 1,000 μm, respectively, are both increasing their presence as delivery systems for diverse treatments. Because of their large surface area-to-volume ratio, NPs and MPs are suited for efficient loading of diverse types of cargos for different applications like drug delivery, immunotherapy, and cancer therapy [58]. Since biopolymers are made of biomolecules, they usually have structures that resemble that of the extracellular matrix and can be easily recognized and metabolized without production of toxic metabolites. For that reason, the development of NPs and MPs from biopolymers has received attention to minimize toxicity concerns associated with synthetic polymers [59].

Natural polymers are mainly made of polysaccharides, polyaminoacids, peptides, proteins, and lipids. These polymers can undergo crosslinking and be used for customized NP design. One advantage is their high resistance to enzymatic degradation or pH changes. Two polysaccharides, chitosan and alginate, stabilized by Ca^2+^ were demonstrated to enhance survival rates of encapsulated phages across the gastrointestinal tract [60–63] but, more importantly, helped to improve the therapeutic outcome of the treatment in broilers infected with *Salmonella* spp., especially in the long term [62]. An interesting in vivo study with chitosan NP for encapsulation of a phage cocktail to treat bacterial diarrhea was done in rats infected with *Salmonella enterica*, *Shigella flexneri*, and *E. coli* [63]. The data were collected by weighing the animals and culturing stool samples through 8 days, comparing among encapsulated bacteriophage cocktail, empty chitosan NPs, cefixime, and control. The results showed that the encapsulated cocktail was an effective treatment for gastrointestinal infections. In vitro release assays confirmed that the chitosan NPs made phages stable at highly acidic pH. In contrast, other studies have confirmed that cationic polymers such as chitosan may reduce phage viability up to 2 or 3 logs [64]. The electrostatic interactions between the polymer and the phage may interfere with phage adsorption process and decrease phage infectivity. As a consequence, phage–polymer interactions must be pre-evaluated before designing a new encapsulation formula. Other examples of polymers that can inhibit phage activity are polylysine, polyarginine, or polyallylamine [65]. Finally, a pH-responsive combination of natural and synthetic polymers (EudragitS100/alginate MPs) was designed for targeted delivery and controlled release of the *E. coli* phage, FelixO1 [66].

### Synthetic polymers

Synthetic NPs and MPs are known for their possibilities in the control of multiple NP features, including size and morphology, and because of their simple preparation. There are different synthesis techniques: emulsification (solvent evaporation) [67], nanoprecipitation [68,69], ionic gelation [70], and microfluidics [71]. This type of NPs makes for good delivery vehicles, being biocompatible and able to carry both hydrophilic and hydrophobic cargos of different molecular weights from small molecules to proteins or vaccines [72–76]. The load can be inside the core of the NP, within the polymer matrix, or attached to the NP surface. Some of the advantages of synthetic polymers are the critical properties of NPs, being loading efficacy and release kinetics, which can be finely tuned by changes in composition, responsivity, stability, or surface charge [77,78]. Attending to structure, there are 2 common possibilities: nano- or microcapsules (polymeric shell with a hollow core) and nano- or microspheres (solid matrix).

Two synthetic polymers are widely used for phage encapsulation delivery systems, with a broad spectrum of therapeutic applications, poly(lactic-co-glycolic) acid (PLGA) and methacrylate-co-methacrylic acid-based (MA-co-MAA). As an example, phage FGS011 was encapsulated in synthetic polymer MPs to treat *Salmonella* sp. in broiler farms. Orally delivered encapsulated phages exhibited higher titers in the cecum of healthy broilers than did nonencapsulated phages, demonstrating an increased capacity for delivering [79]. In another work, phages targeting *S. aureus* and *P. aeruginosa* were successfully encapsulated in PLGA MPs in a freeze-dried formulation. Their size, composition, and release profile suggested them as an interesting treatment for pulmonary infections [80]. Another possibility for the treatment of *P. aeruginosa* in lung infections was proposed using phages deposited on the surface of porous PLGA MPs for nebulized administration [81]. The results of in vivo administration of phage-loaded MPs in a murine model showed distribution through the lungs without any safety issues observed. More importantly, phage–NPs reduced the counts of *P. aeruginosa* in the lungs by an order of magnitude compared with a nonencapsulated phage treatment, with a 100% survival rate. Interestingly, these results also translated to mutant mice that mimic human cystic fibrosis, which includes a more severe disease when infected with *P. aeruginos*a [82,83]. The treatment with encapsulated phages reduced the counts of bacteria near the experimental detection limit. Last, the formulation was evaluated in clinical isolates from lung infections in cystic fibrosis patients, demonstrating the capacity of killing the bacteria in the lung infection isolates in over 80% of cystic fibrosis clinical strains. The results demonstrated the potential of polymeric NPs as vehicles for targeted phage therapy in lung infections caused by *P. aeruginosa* in both acute lung infections and cystic fibrosis-related infections.

Another application of encapsulation can be found in the design of phage-based vaccines. An approach to this was obtained using fd filamentous phage, as a model for an antigen display system, capable of inducing a strong immune response (both innate and adaptive) [84,85]. An engineered fd phage that expressed the ovalbumin (OVA) antigen was encapsulated into PLGA MPs and used as a vaccine model candidate [86]. The results revealed that phage maintained its integrity and immunogenic capacity after encapsulation, presenting encapsulation as a potential tool for the development of phage-based vaccines.

Unfortunately, one shortcoming of synthetic polymers is the involvement of organic solvents in certain synthesis steps, which may lead to phage inactivation and decrease the phage concentration over time. To overcome this, it is necessary to select the solvent wisely. Every pair of phage–solvent must be evaluated for possible incompatibilities to assure viability and therapeutic efficacy after encapsulation [87].

### Liposomes

The encapsulation of phages in a lipid-base formulation has been extensively studied for the treatment of gastrointestinal, respiratory, and intracellular pathogens [56,88]. Among the lipid-base systems, liposomes are of particular interest. Liposomes are spherical NPs formed by a lipid bilayer of phospholipids that surrounds an aqueous cavity. Because of the dual nature of the liposome, hydrophilic and amphiphilic molecules can be encapsulated into the polar core, while hydrophobic substances can be entrapped into the lipid membrane. The main asset of liposomes is their composition, which provides them with high biocompatibility, being also easily produced. The principal methods for liposome production are the thin-film hydration method [89–93], gel-assisted rehydration [94], inverse emulsion [94], and microfluidics [95,96]. Thin-film methods are the most used in laboratory settings because of the simplicity and cost-effectiveness of the procedure. However, they are not the best suited for an industrial scale-up due to a lack of reproducibility and little homogeneity between the particles obtained. The use of microfluidics is key for the production of low polydisperse (nonheterogeneous) NPs in a precisely controlled and reproducible manner.

Liposomes can be structurally divided into unilamellar or multilamellar, and by means of composition changes, some parameters such as surface charge can be adapted, having a further influence on delivery or pharmacokinetics [97]. Despite their successful use for drug or nucleic acid delivery, liposomes present a limitation when it comes to phage therapy since their small size can be a limiting factor for the encapsulation of large phages. Liposomal size can be controlled to a certain point by sonication, by extrusion through membranes, or by using microfluidics, but eventually, they can adhere to each other and can undergo fusion, losing their original size. The production of liposomes that are able to retain their size (i.e., without aggregation or fusion) adopts special importance if it were to be used for systemic administration or in the treatment of intracellular pathogens. Other factors that must be considered before the implementation of liposomes in phage therapy are stability and loading efficacy. Furthermore, the size of the encapsulated phages may have an effect on the final size of the liposome. This was demonstrated after the encapsulation of 2 *Pseudomonas* spp. phages, PEV2 (podovirus, diameter 65 nm) and PEV40 (myovirus, diameter 220 nm), into liposomes. Despite using the same synthesis conditions, the average size of the resulting liposomes was slightly smaller for PEV2 (135 to 218 nm) than for PEV40 (261 to 448 nm), a difference that correlates with phage particle sizes [95].

One fundamental property of liposome encapsulation is that it can facilitate phage access into phagocytic cells and infection of intracellular pathogens, like *Mycobacterium tuberculosis*, *Listeria* spp., *Salmonella* spp., and *Staphylococcus* spp. Moreover, they serve as a platform to adhere to mucous surfaces where bacteria may reside (e.g., in respiratory or gastrointestinal infections), improving also phage diffusion ability. Focusing on gastrointestinal infections, a common problem arises from the short residence time of orally ingested phages, usually aggravated by symptoms such as diarrhea; thus, liposomes can be a good approach for elevating residence times through mucoadhesion [97]. Another limitation in the oral administration of phages is their poor stability under the acidic pH of the stomach [11]. Again, nanoencapsulation in liposomes can overcome these potential drawbacks. For example, chickens infected with *Salmonella* spp. were treated with a liposome-encapsulated cocktail. The results showed increased efficacy and prolonged protection of encapsulated phages when administered orally to broiler chickens. After 72 h of administration, encapsulated phages were detected in 38.1% of the treated animals versus 9.5% of detection in chickens administered with free phages [89].

Liposome particles with cholesterol and PEG also help phages to undergo transcytosis in the intestinal epithelium. On the one hand, the small size of the liposomes is suitable for cellular uptake. On the other hand, the presence of cholesterol and PEG can protect against clearing by the reticuloendothelial system, improving circulation time. The lipid nature and positive charge of the liposomes can favor mucoadhesion and hence cellular uptake. As an example, the concentration of phage UAB_Phi20 of *S. enterica* in the stomach of mice was significantly higher when administered encapsulated, even 6 h after the administration. More importantly, the phage titer was not reduced over that time. These findings suggested that liposome encapsulation facilitated adherence to the intestinal membrane, diffusion across the intestinal epithelium, and prolonged presence of encapsulated phages in the stomach [90]. Another example of liposome encapsulation was done with KPO1K2 *Klebsiella pneumoniae* phage, showing enhanced retention of encapsulated phages in mice blood, kidney, liver, and spleen compared to nonencapsulated phages [91].

In spite of many advantages, one of the major threats to phage therapy is the development of an immune response to phages, which can induce the production of phage-neutralizing Abs. On repeated administration, the effectiveness of phage therapy was found to decrease as a consequence of the Ab-mediated inactivation of phages [98,99]. The use of liposomes as a delivery system permits the masking of phages, avoiding immune responses. This has been tested using the KPO1K2 phage. Free phage formulation and liposome-encapsulated phages were tested in vitro in a phage neutralization test. After 3 h of incubation with antiserum containing the phage-specific Abs, free phages lost viability, while encapsulated phages maintained their original titer constant [100]. When bacteria were added to the medium containing both formulations of phages and Abs, it was verified that free phages lost their killing capacity because of an increase in bacterial population 3 h later, facing the 2-log decrease of bacterial burden when added to entrapped phages. Encapsulation in liposomes not only blocked the Ab-mediated phage inactivation but also facilitated phage delivery inside macrophages, leading to the killing of intracellular *K. pneumoniae*. These results suggest liposomes as an interesting tool to counteract some deficiencies of phage therapy, Ab-mediated neutralization, and the inability of phages to target intracellular pathogens.

Encapsulation of bacteriophage cocktails into liposomes was also evaluated for wound healing. In this sense, burn wound infections of *K. pneumoniae* in mice were treated with a phage cocktail. The bacterial counts were lower in the skin, liver, and blood when treated with a liposome-encapsulated cocktail, and consistently, the infection process was shorter in the animals with this treatment [92]. The improved effect of the encapsulated cocktail may be provided by a longer residence time in vivo over the nonencapsulated phages. Although free phages were capable of rescuing all mice from death when applied at the start of infection, only liposome entrapped cocktail was able to avoid the death of 100% of mice after 24 h of delayed treatment. Promising results were also achieved by encapsulation of MR-5 and MR-10 phages as a cocktail in the reduction of infection caused by *S. aureus*, where enhanced availability at the infection site was observed compared to nonencapsulated cocktail, and wound closure was 3 days faster in mice treated with liposome-encapsulated phages [93].

In another study, microfluidics was used to encapsulate 2 model phages (*E. coli* T3 and *S. aureus* K phage) in liposomes [96]. In the case of T3, phage aggregation was observed even at low concentrations of phage, affecting encapsulation yield. For phage K, a great number of viral particles were attached to the surface of the liposome instead of being entrapped on the inside due to interactions between the phage and the lipid bilayer. After the inactivation of the external phages, the real encapsulation yield was determined. The authors reflect that previous research may have overestimated phage encapsulation yields owing to this problem. It is important to take this into account when aiming for precise dosing and delivery, as externally adhered phages may suffer inactivation caused by external factors in the same way as free phages, for example, when exposed to gastric acid in the stomach. As a result, the phage concentration at the site of infection could be lower than expected.

Niosomes and transferosomes were also studied as an alternative to liposomes for bacteriophage encapsulation [101]. Their structure resembles that of liposomes; they can be unilamellar or multilamellar, but the composition slightly differs from that of liposomes. Niosomes are formed by amphiphilic molecules other than phospholipids (i.e., nonionic surfactants) [102]. Transferosomes are a combination of phospholipids and nonionic surfactants. In the study, the encapsulation potential of the 3 systems (liposomes, niosomes, and transferosomes) was evaluated using the bacteriophage phiIPLA-RODI [101]. The encapsulation was effective in all cases, with yields ranging from 62% to 98%. The long-term stability of encapsulated phages was also tested, demonstrating that they maintained infectivity after 6 months of storage at 4 °C.

## Conclusions

The emergence of MDR bacteria is nowadays considered a major threat that requires multidisciplinary approaches to develop new control strategies. Under this scenario, phage-based nanotechnology can be a very promising and efficient alternative to overcoming antimicrobial resistance. The use of phage-linked NPs can be exploited in both prevention, diagnosis, and treatment, increasing the chances of eradication of pathogenic bacteria. Early detection of bacterial strains with increased sensitivity and selectivity could be affordable at competitive costs and easily implemented in the clinic since costly equipment is not required to perform these techniques. On the other hand, the combination of phages with NPs can improve photothermal and targeted PDTs, and can be useful for the treatment of infections associated with implants and medical devices, especially in orthopedics, dentistry, and urology. In addition, phage encapsulation provides greater stability, increased bioavailability, avoidance of loss of activity, or rapid elimination from the body, as well as allowing targeted delivery. For all these reasons, it is important to design new types of NPs for phage delivery, combining the advantages of natural polymers and liposomes (bioavailability, biocompatibility) and synthetic polymers (stability, encapsulation efficiency, diversity). Improved production processes will ensure the reproducibility and scale-up of bacteriophage-based nanomaterials to meet industrial production requirements. Although there is still a long way to go, especially at the regulatory level, phage-based nanotechnology is in the spotlight and future research in the field will improve the development of new antimicrobial products.

## Data Availability

Data sharing is not applicable to this article as no new data were created or analyzed in this study.
